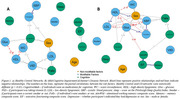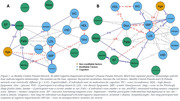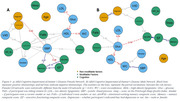# Sex‐specific relationships among risk factors in those with Mild Cognitive Impairment or Alzheimer’s disease and healthy controls

**DOI:** 10.1002/alz.092923

**Published:** 2025-01-09

**Authors:** Brittany Intzandt, Joel Ramirez, Benjamin Lam, Mario Masellis, Christopher J. M. Scott, Gillian Einstein, Louis Bherer, Sandra E Black, ONDRI Researchers; CCNA Researchers

**Affiliations:** ^1^ Sunnybrook Research Institute, Toronto, ON Canada; ^2^ Sandra Black Centre for Brain Resilience and Recovery, Sunnybrook Research Institute, Toronto, ON Canada; ^3^ University of Toronto, Toronto, ON Canada; ^4^ Heart and Stroke Foundation Canadian Partnership for Stroke Recovery, Toronto, ON Canada; ^5^ LC Campbell Cognitive Neurology Research Unit, Sunnybrook Research Institute, Toronto, ON Canada; ^6^ Hurvitz Brain Sciences Research Program, Sunnybrook Research Institute, Toronto, ON Canada; ^7^ Toronto Dementia Research Alliance, Toronto, ON Canada; ^8^ Campbell Cognitive Neurology Research Unit, Sunnybrook Research Institute, Toronto, ON Canada; ^9^ Cognitive and Movement Disorders Clinic, Sunnybrook Health Sciences Center, Toronto, ON Canada; ^10^ Faculty of Medicine, University of Toronto, Toronto, ON Canada; ^11^ Hurvitz Brain Sciences Program, Sunnybrook Research Institute, Toronto, ON Canada; ^12^ Canadian Partnership for Stroke Recovery, Toronto, ON Canada; ^13^ Rotman Research Institute, Toronto, ON Canada; ^14^ Université de Montréal, Montréal, QC Canada; ^15^ Montreal Heart Institute, Montréal, QC Canada; ^16^ Division of Neurology, Department of Medicine, University of Toronto, Toronto, ON Canada; ^17^ LC Campbell Cognitive Neurology Research Unit, Sunnybrook Research Institute, University of Toronto, Toronto, ON Canada

## Abstract

**Background:**

Dementia incidence is projected to significantly increase, posing unique challenges to healthcare systems. Identifying non‐modifiable and modifiable risk factors (RF) is crucial, including sex‐specific factors, given the higher prevalence among females (60%). Here, we employed a network analysis to examine prominent RF in healthy controls compared to those with cognitive decline (CD), as well as the interrelationships and interactions of RF on CD. Additionally, sex‐specific networks were compared to identify unique RF and interactions present among sex.

**Method:**

Healthy controls and CD individuals (mild cognitive impairment and Alzheimer’s dementia) were included from the Ontario Neurodegenerative Initiative and Canadian Consortium for Neurodegeneration in Aging (n = 339 total; 52% female; 72% CD). Non modifiable RF (e.g., age), modifiable RF (e.g., Framingham RF) and cognitive outcomes (e.g., executive functioning) were included in network modeling. Sex‐specific networks were created within the CD group and compared, as was between CD and healthy controls. Relationships among RF present in CD were identified and the strength. Nodes represented RF and edges are the pairwise dependency between RF, node centrality was investigated for the relative importance of each RF in the network.

**Result:**

Healthy controls and CD had statistically different networks (M = 0.536; p = 0.02), and the CD network had greater connectivity (S = 2.69; p = 0.005)[Figure 1]. Male and female networks were statistically different within CD (M = 0.432; p = 0.027), and the male’s network had statistically greater connectivity than the females with CD (S = 1.24; p = 0.049)[Figure 2]. Within females, the CD had significantly greater connectivity (S = 0.90; p = 0.03)[Figure 3] than healthy controls and no difference in males (p > 0.05).

**Conclusion:**

Our findings reveal unique sex‐specific network patterns of RF for CD, which further underscores the need for sex‐disaggregated analyses. The observed differences in heightened connectivity of typically studied RF in males, highlights a potential gap in the understanding of sex‐specific RF for Alzheimer’s. Future work should incorporate biomarkers, such as neuroimaging, to further comprehend sex‐specific RF for CD and to create the framework for precision medicine in targeting sex‐specific RF for CD.